# Metal Oxide-Derived MOF-74 Polymer Composites through Pickering Emulsion-Templating: Interfacial Recrystallization, Hierarchical Architectures, and CO_2_ Capture Performances

**DOI:** 10.1021/acsami.3c01796

**Published:** 2023-03-30

**Authors:** Nika Vrtovec, Sarah Jurjevec, Nataša Zabukovec Logar, Matjaž Mazaj, Sebastijan Kovačič

**Affiliations:** † National Institute of Chemistry, Hajdrihova 19, 1000 Ljubljana, Slovenia; ‡ University of Nova Gorica, Vipavska 13, 5000 Nova Gorica, Slovenia

**Keywords:** Pickering HIPEs, ROMP, polyHIPEs, MOF-74, secondary recrystallization, CO_2_ capture

## Abstract

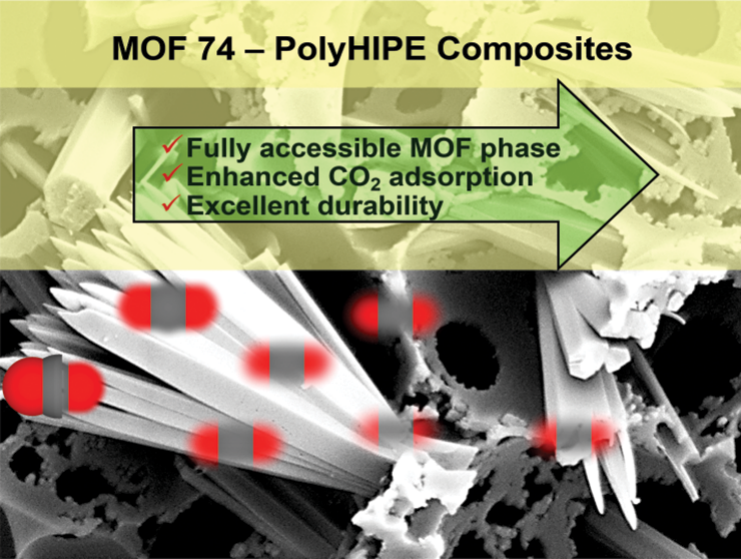

Currently, metal–organic framework (MOF)–polymer composites are attracting great interest as a step forward in making MOFs a useful material for industrially relevant applications. However, most of the research is engaged with finding promising MOF/polymer pairs and less with the synthetic methods by which these materials are then combined, albeit hybridization has a significant impact on the properties of the new composite macrostructure. Thus, the focus of this work is on the innovative hybridization of MOFs and polymerized high internal phase emulsions (polyHIPEs), two classes of materials that exhibit porosity at different length scales. The main thrust is the in situ secondary recrystallization, i.e., growth of MOFs from metal oxides previously fixed in polyHIPEs by the Pickering HIPE-templating, and further structure-function study of composites through the CO_2_ capture behavior. The combination of Pickering HIPE polymerization and secondary recrystallization at the metal oxide–polymer interface proved advantageous, as MOF-74 isostructures based on different metal cations (M^2+^ = Mg, Co, or Zn) could be successfully shaped in the polyHIPEs’ macropores without affecting the properties of the individual components. The successful hybridization resulted in highly porous, co-continuous MOF-74–polyHIPE composite monoliths forming an architectural hierarchy with pronounced macro-microporosity, in which the MOF microporosity is almost completely accessible for gases, i.e., about 87% of the micropores, and the monoliths exhibit excellent mechanical stability. The well-structured porous architecture of the composites showed superior CO_2_ capture performance compared to the parent MOF-74 powders. Both adsorption and desorption kinetics are significantly faster for composites. Regeneration by temperature swing adsorption recovers about 88% of the total adsorption capacity of the composite, while it is lower for the parent MOF-74 powders (about 75%). Finally, the composites exhibit about 30% improvement in CO_2_ uptake under working conditions compared to the parent MOF-74 powders, and some of the composites are able to retain 99% of the original adsorption capacity after five adsorption/desorption cycles.

## 1 Introduction

The development of carbon capture storage and utilization (CCSU) technologies is becoming a necessity in order to approach the targets of sustainability and hamper the CO_2_ emission growth. CO_2_ capture on solid adsorbents represents one of the most promising alternatives that can overcome highly demanding requirements for energy consumption and environmental risks associated with the conventionally used amine scrubbing process.^1^ Metal–organic frameworks (MOFs) are being intensively investigated for CCSU applications, as the physical and chemical properties can be tailored through rational design, enabling optimization of CO_2_ capture performance.^2−5^ In the previous decade, most attention has been paid to the fundamental study of different synthesis approaches, their optimization, and framework functionalization in order to improve MOF capabilities/performance for the selected application, while recently, the research is more focused also on the practical use of MOFs. However, progress in the application of MOFs in industrially relevant processes is severely limited by their form factor (i.e., powder). As a further development of MOFs’ rational design, shaping powder products into various macrostructures is a step forward in bringing MOFs closer to a useful material. Various approaches have been introduced for formation of thin films, membranes, granules, tablets, pellets, or monoliths.^6−8^ To improve application-specific properties and handling, forming MOFs into monolithic macrostructures is particularly important because they offer large geometric surface area, low-pressure drop, and low mass transfer resistance and are easy to handle and scale up.^9−13^ Although significant advantages have been achieved with monolithic macrostructures, the processability and physical robustness in pure MOF monoliths remain a challenge. A convenient strategy to improve these shortcomings is to combine MOFs with easily processable and mechanically robust organic polymers forming MOF–polymer composite materials.^14,15^

The concept of mixing MOFs and polymers is not new, as there are numerous MOF/polymer pairs that have been combined to form various composites, such as mixed matrix membranes, mixed material fibers, or covalent composite materials, to name a few.^16,17^ Although various synthesis strategies have been developed to achieve a form factor with distinct polymer characteristics, aggregation-related defects and brittle mechanical properties are still a problem, especially at higher MOF loadings, and furthermore, the liquid polymer precursors or the molten polymer can enter into and clog the micropores of the MOFs, reducing composite functionality. Therefore, it is an interesting yet challenging idea to develop highly porous co-continuous MOF–polymer composite monoliths in which microporous MOFs homogeneously fill a macroporous polymer framework. Ideally, the resulting structured materials will exhibit hierarchical pore architecture with high and unobstructed mass diffusion, will be easy to handle, and physically robust. Here, we present a simple and inexpensive synthetic strategy that combines Pickering emulsion-templating and metal oxide-derived MOFs via secondary recrystallization to fabricate such co-continuous hierarchical porous monolithic composites.

Emulsion-templating, using high internal phase emulsions (HIPEs) as a structural template, has recently gained particular attention as a technique for preparing macroporous polymer matrices called polyHIPEs (PHs).^18^ PHs are typically single-piece polymer foams characterized by unique 3D-interconnected microcellular morphology and tunable mechanical properties, which can be based on different chemical compositions. In addition, various organic–inorganic composites (hybrids) have also been investigated by HIPE-templating.^19,20^ PH composites are obtained from nanoparticle-stabilized HIPEs, a process called Pickering-stabilization, which usually uses metal-oxides (MO) of various compositions. However, we and others have shown microporous MOFs^21−29^ and zeolites^29^ having suitable surface properties for stabilizing HIPEs and forming PH composites. MOF–PH composites were also formed by solvothermal MOF growth on the voids surface in preformed PHs.^30−33^ In practice, it appears that the form factor and physical robustness can be solved by using emulsion templates to obtain MOF–PH composites. However, micropore clogging (Pickering stabilization strategy) or low loading of the MOF phase (in situ MOF growth strategy) is still shortfalls which severely affects the performance of these composites in certain applications such as CO_2_ capture or catalysis. In response, we have developed a new seeding strategy using MO nanoparticles as sacrificial metal precursors and obtained MOF–PH composites directly from MO–PH macrostructures that revealed enhanced MOF loading and micropore accessibility.^28^

As a further evolution of this MO-to-MOF secondary recrystallization approach for the fabrication of MOF–PH macrostructures, we have developed in this work the first co-continuous MOF-74–PH composites in which the microporous MOF-74 phase homogeneously fills or coats a PH macroporous structure throughout the monolith, creating an architectural hierarchy. MgO, ZnO, and Co_3_O_4_ are first fixed in the PH void walls and then serve as a source of metal cations (M^2+^) that react with the 2,5-dihydroxy-1,4-benzenedicarboxylate ligand (DHBDC) at the MO–polymer interface to form MOF-74 isostructures. PHs prepared by ring-opening metathesis polymerization (ROMP) exhibit favorable mechanical properties and have been used as separators in Li-ion batteries,^34^ as oxygen scavengers,^35^ or as precursors for the preparation of macroporous carbons by carbonization.^36^ In particular, the poly(dicyclopentadiene) (PDCPD) PH matrix has been shown to be advantageous in the preparation of MO–PH^37,38^ and MOF–PH^21,28^ nanocomposites, as it exhibits properties such as high toughness and stiffness, high temperature and corrosion resistance, and excellent chemical resistance,^39^ and thus seems to be the perfect choice for our MO-to-MOF secondary recrystallization synthesis. On the other hand, the MOF-74 prototype was selected because it has a large specific surface area and a high density of open metal sites that allow exceptionally high CO_2_ uptake under ambient conditions, which is a favorable property for our CO_2_ capture application.^3,40,41^ The influence of different Pickering systems, i.e., water-in-dicyclopentadiene (W/O) HIPEs stabilized by MgO, ZnO, or Co_3_O_4_, on the MO/MOF content in the PH structure was investigated. Finally, the porous and morphological properties of MOF-74-based PH composites are discussed and CO_2_ capture performance is evaluated.

## 2 Experimental Section

### 2.1 Synthesis of Materials

#### 2.1.1 MOF-74 Powders

A series of powdered MOF-74 isostructures were synthesized using metal salts or metal-oxides as the source of metal cations (M^2+^ = Mg, Co, or Zn) and the 2,5-dihydroxy-1,4-benzenedicarboxylate (DHBDC) as the organic linker, which were solvothermally reacted in various solvents (see details in the Supporting Information). Briefly, Zn(NO_3_)_2_·6H_2_O, Co(ac)_2_·4H_2_O, and Mg(NO_3_)_2_·6H_2_O or ZnO, Co_3_O_4_, and MgO were dissolved and then DHBDC was added with constant stirring. The reaction mixture was transferred to a Teflon-lined autoclave and heated for various periods of time (see the Supporting Information). After cooling to room temperature, MOF-74 powders were obtained by filtration.

#### 2.1.2 MOF-74-Based PH Composites

Initially, PH composites containing metal oxides were prepared by Pickering HIPE-templating. Water-in-oil (W/O) HIPEs were stabilized by oleic acid coated ZnO, Co_3_O_4_, or MgO and PDCPD–PH composites were synthesized (see details in the Supporting Information). For recrystallization of metal oxide-based PHs, DHBDC was dissolved in a suitable solvent and a piece of PH composites, i.e., containing either ZnO, Co_3_O_4_, or MgO nanoparticles, was added to the mixture. The reaction mixture was then transferred to a Teflon-lined stainless-steel autoclave and heated at 150 °C for 48 h. After solvothermal treatment, the recrystallized composites were rinsed with acetone and dried under ambient conditions.

### 2.2 Characterization

Powder XRD data of all synthesized and recrystallized M-MOF-74 were collected on a PANalytical X’Pert PRO diffractometer using CuKα radiation (λ = 1.5418 Å) at room temperature in an angular range of 5–60° (2θ) with a step size of 0.033° per 100 s using a fully opened 100 channel X’Celerator Detector. Thermal stability of synthesized and recrystallized samples, mass fractions of incorporated MOs within MO-based PH composite and recrystallized MOF-74 isostructures within MOF-74-based PHs were determined by thermogravimetric measurements performed on a TA Instruments Q5000. The measurements were carried out in airflow of 10 mL/min, by heating samples from 25 to 700 °C at the rate of 5 °C/min. The morphologies of the composite samples were examined by scanning electron microscopy using Zeiss FEG SEM SUPRA 35 VP. All sorption measurements and kinetic studies were performed on the manometric gas analysis system HTP-IMI Hiden Isochema Inc. Before the measurements, MOF-74 powders were activated under vacuum at 230 °C for 12 h, while MOF-74-based PH composites were activated under vacuum at 170 °C for 12 h. Brauner–Emmett–Teller (BET) specific surface areas were calculated from CO_2_ isotherms performed up to relative pressure *p*/*p*_0_ = 0.9 and temperature 273 K. The CO_2_ sorption capacities and kinetic profiles of all samples were performed at a pressure of 1 bar and a temperature of 25 °C. Measurements of working capacity and adsorbent regenerations were performed in the temperature range between 25 and 150 °C and pressure up to 1 bar. All sorption results for MOF-74-based PH composites were calculated based on the amount of incorporated MOF.

## 3 Results and Discussion

### 3.1 Synthesis and Morphological Properties: MO-to-MOF Secondary Recrystallization

**Figure 1:**
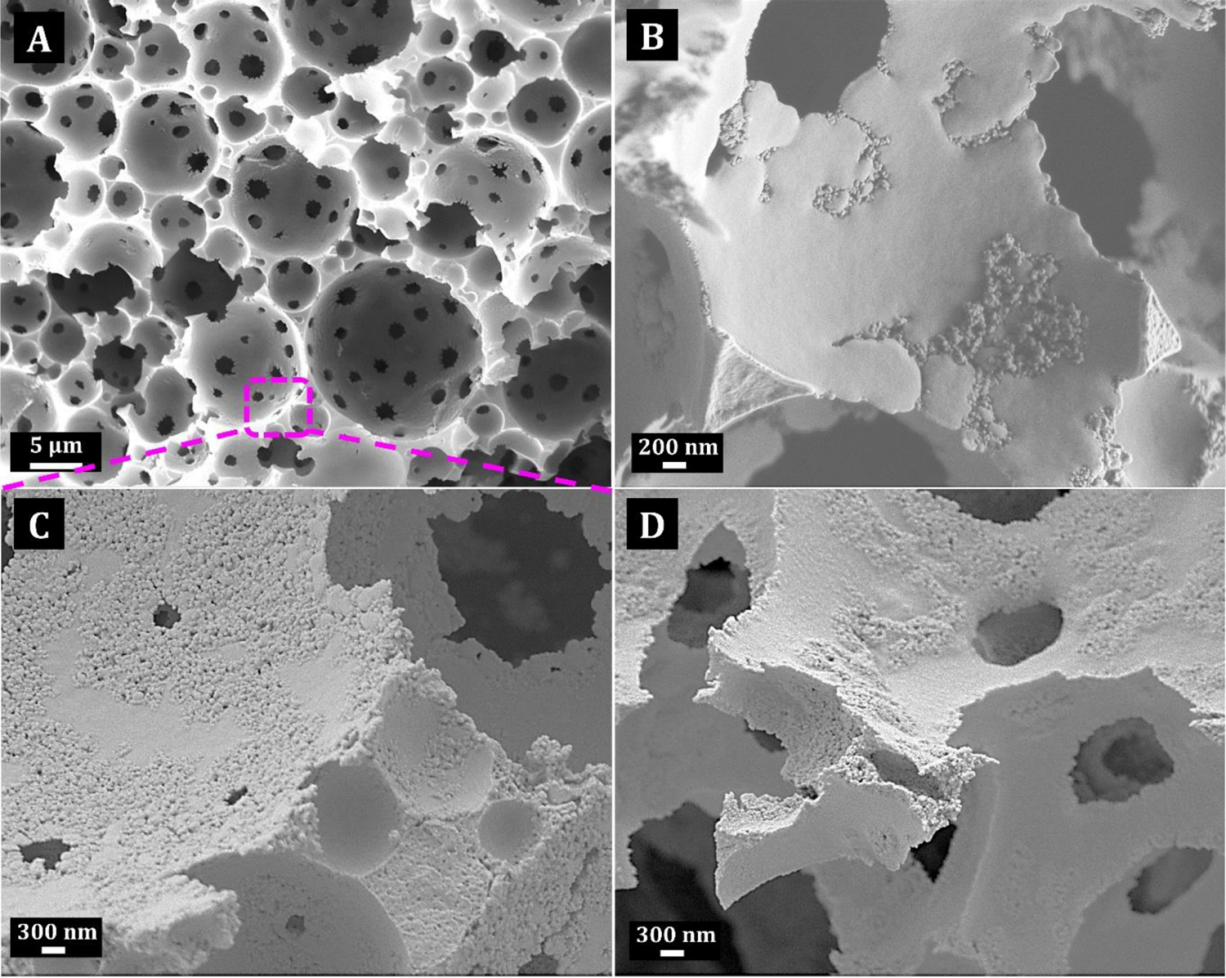
SEM micrographs of ZnO-PDCPD PH composite (A) and high-magnification SEM images of MgO-PDCPD PH sample (B), ZnO-PDCPD PH sample (C), and Co_3_O_4_-PDCPD PH sample (D).

MOF-74-based polyHIPE (PH) composites were prepared using the Pickering HIPE-templating technique. In this process, metal-oxide nanoparticles (MO) are first embedded in the PH matrix and then used as sacrificial metal precursors that react solvothermally with the organic linker, i.e., 2,5-dihydroxyterephthalic acid (DHBDC), to form a MOF in PH. To demonstrate this, we used different MO, i.e., ZnO, Co_3_O_4,_ and MgO, embedded in the PDCPD PH matrix. Initially, PDCPD PHs were prepared from water-in-DCPD (W/O) Pickering HIPE systems, and for this purpose, different combinations of MOs and surfactant (PluronicL-121) were tested as stabilizers. Formulations that contained at least 5 wt % surfactant relative to DCPD proved to be the most promising, regardless of the amount of MOs.^37,38^ The MOs must first be surface modified with oleic acid (OA) to improve wettability with the DCPD (continuous) phase and the best stabilization of the water-in-DCPD HIPEs was achieved when the MO were surface functionalized with ∼12 wt % OA (determined by TG analysis; Figure S1). The stable HIPEs were finally cured by ROMP, and the obtained rigid monoliths were Soxhlet extracted and vacuum dried. In optimizing the synthesis, several W/O Pickering HIPEs with different MO contents (between 10 and 30 wt %) were prepared, and a content of 20 wt % proved to be the best compromise between the highest loading, good HIPE stability, and mechanical integrity of the final PH composites. The good moldability of the prepared Pickering HIPEs was already evident upon visual inspection, i.e., gel points were reached within a few seconds without evoked phase separation, so that evaluation of the PH composite with SEM only confirmed the typical polyHIPE structure (Figure 1). All the MO–PH composites have a highly interconnected, porous structure typical of PHs. Voids in PDCPD-based PH composites were about 5 ± 3 μm in diameter (Figure 1A). High-magnification SEM images also show that there is a certain amount of MO visible on the surface of the voids (Figure 1B–D).

The results of the thermogravimetric examination of the PH composites indeed confirmed the presence of MO in PH matrices. First, the thermal stability of pure PDCPD PH was investigated, and it was found that the onset of thermal decomposition starts at about 200 °C, while the PDCPD matrix is completely decomposed as the temperature further increases to about 480 °C, as shown by the TGA analysis (Figure S3). Then, the residual mass was evaluated when PH composites were heated in the airflow at 800 °C and was found about 18 wt %. Since the PH matrix completely decomposes in the air flow already at 480 °C, these residual masses determined from the TGA of the PH composites can be directly referred to MO.

**Figure 2:**
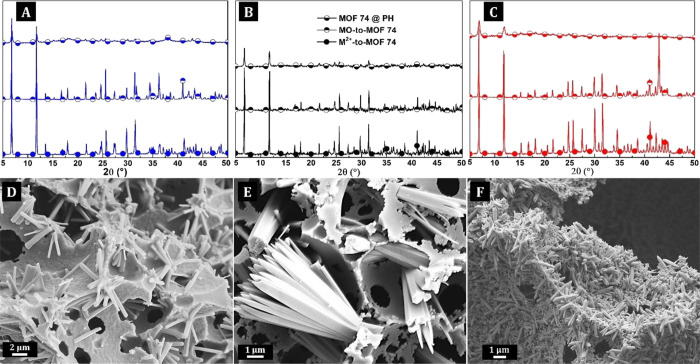
XRD patterns of Zn-based materials (A), Co-based materials (B), and Mg-based materials (C), with MOF74 in PH (upper patterns), MO-to-MOF-74 (middle patterns), and M^2+^-to-MOF-74 (bottom patterns). SEM micrographs of MOF-74-derived composite PH based on Zn- (D), Mg- (E), and Co-based MOF-74 (F).

Prior to recrystallization, a series of powder MOF-74 isostructures were synthesized using metal salts (direct crystallization) or MO (secondary recrystallization) as a source of metal cations (M^2+^ = Mg, Co, or Zn) that solvothermally react with organic linker (DHBDC). In all cases, the optimized synthesis conditions led to the successful crystallization of the Zn-, Mg-, and Co-based MOF-74 isostructures, as shown by XRD analysis, which exhibit 1D hexagonal aligned channels of approximate 1 nm in diameter, with high density of unsaturated metal sites ideal for the selective adsorption of polar gases. The XRD patterns depicted in Figure 2A–C reveal that the MOF-74 crystalline phase is present in all samples. However, some unreacted oxide phases were still present in the MgO- and ZnO-derived MOF-74 products (Figures S4 and S5).

The next step was the MO-to-MOF recrystallization directly in the PH matrices following the synthesis conditions optimized for the powders. First, the morphology of MOF–PH composites was visualized by the SEM analysis, and images are shown in Figure 2D–F. In all cases, the typical 3D-interconnected PH morphology was fully preserved during the recrystallization process (Figure S7). Different from the MO–PH samples, these composites contain elongated crystallites in the voids, exhibiting a typical MOF-74 topology. The presence of the MOF-74 isostructures was corroborated by the XRD analysis. It seems that complete MO-to-MOF conversion was achieved in all cases since no MO residues were found in the PHs (Figure 2A–C). However, the detection of the potential peaks belonging to the MO phases may be overlaid with the increased background of the XRD patterns due to the amorphous polymer in the composite. Therefore, the TG analysis was used to additionally verify the extent of the MO-to-MOF conversion and thus the recrystallization efficiency while determining the MOF content in the PH composites (Table 1, Figures S8–S10). Residual masses increased from 16 to 18 wt % in the MO composite PHs to 23 to 25 wt % in the MOF composite PHs, indicating that the MO-to-MOF conversion yields ranged from 55 to 77% (for the details see the Supporting Information).

**Table 1:** MO, MOF-74 Content, and Recrystallization Yields in the PDCPD PH Matrix

type of MOF-74 isostructure	MO content (wt %)	MOF-74 content (wt %)	recrystallization yield^a^ (%)
Zn	18	23	74
Mg	16	24	55
Co	17	25	77

a MOF-74 recrystallization yields from MO@PDCPD PH precursors.

**Figure 3:**
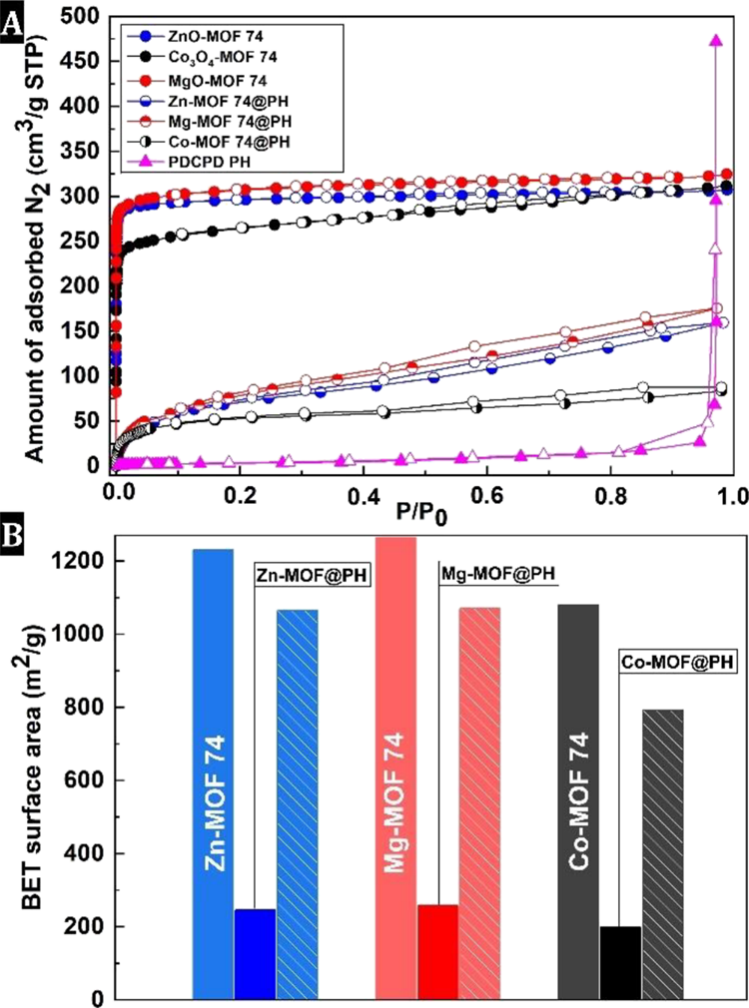
N_2_ isotherms of reference materials (MOF-74 powder and PDCPD PH) and PH composites (A) *S*_BET_ values for MOF-74 powder and MOF-74@PH composites (B). Patterned columns show the calculated *S*_BET_ of MOF-74@PH composites based on the MOF content in the PH matrices (determined by TGA).

Porous properties and associated specific surface areas were further analyzed by nitrogen adsorption–desorption measurements. The N_2_ isothermal data for the reference materials, i.e., MO-derived MOF-74 powders and PDCPD PH matrix, were evaluated and compared with the PH composites (Figure 3). The PDCPD PH exhibits neither accessible microporosity nor mesoporosity. Instead, a steep increase in N_2_ sorption uptake at *P*/*P*_0_ ≈ 1 indicates the presence of macropores with specific surface areas (*S*_BET_) of 0.5 m^2^ g^–1^. On the other hand, the MOF-74 powders exhibited type I isotherms in all cases (typical of microporous materials) with *S*_BET_ of 1231, 1264, and 1080 m^2^ g^–1^ for Zn-, Mg, and Co-based MOF-74 isostructures, respectively. However, the fixation of MOF-74 in a macroporous PDCPD PH framework led to a hierarchically porous system with pronounced macro-microporosity. The shape of the isotherms of PH composites is reminiscent of that of macroporous PHs with a steep increase in N_2_ sorption uptake at *P*/*P*_0_ ≈ 1, and it is also very similar to that of microporous MOFs following the type I isotherm, with a significant increase in N_2_ uptake in the *P*/*P*_0_ range up to 0.1 (Figure 3A).

The macro-microporous structure reflects in *S*_BET_ of 245, 257, and 198 m^2^ g^–1^ for Zn-, Mg-, and Co-based PH composites, respectively. When the *S*_BET_ calculation includes only the net MOF-74 phase (according to TGA) without the polyHIPE matrix, the surface areas of composites are very close to those of the parent MOF-74, i.e., 1068, 1105, and 790 m^2^ g^–1^ for Zn-, Mg-, and Co-based MOF–PH composites, respectively (Figure 3B). To take advantage of MOFs fixed-in polymer matrices, e.g., for separation or catalysis purposes, an accessible micropore structure is essential. The accessibility of MOF-74 micropores to gases was, therefore, evaluated for all MOF-based PH composites and compared to parent MOF-74 powders by analyzing the N_2_ sorption isotherms. Considering the content of MOF phase in the PH matrices (details in SI), the accessibility was then calculated as the ratio between the *S*_BET_ values of the parent MOF powders and MOFs fixed-in polymer matrices. It was found that about 75–87% of the PH composite’s microporosity is accessible in the structure, indicating unobstructed gas diffusivity to the MOF-74 phase. Thus, it appears that our MO-to-MOF recrystallization approach is indeed a promising technique for the preparation of polymer-MOF mixed materials with highly accessible MOF phase.

### 3.2 CO_2_ Uptake Performances

**Figure 4:**
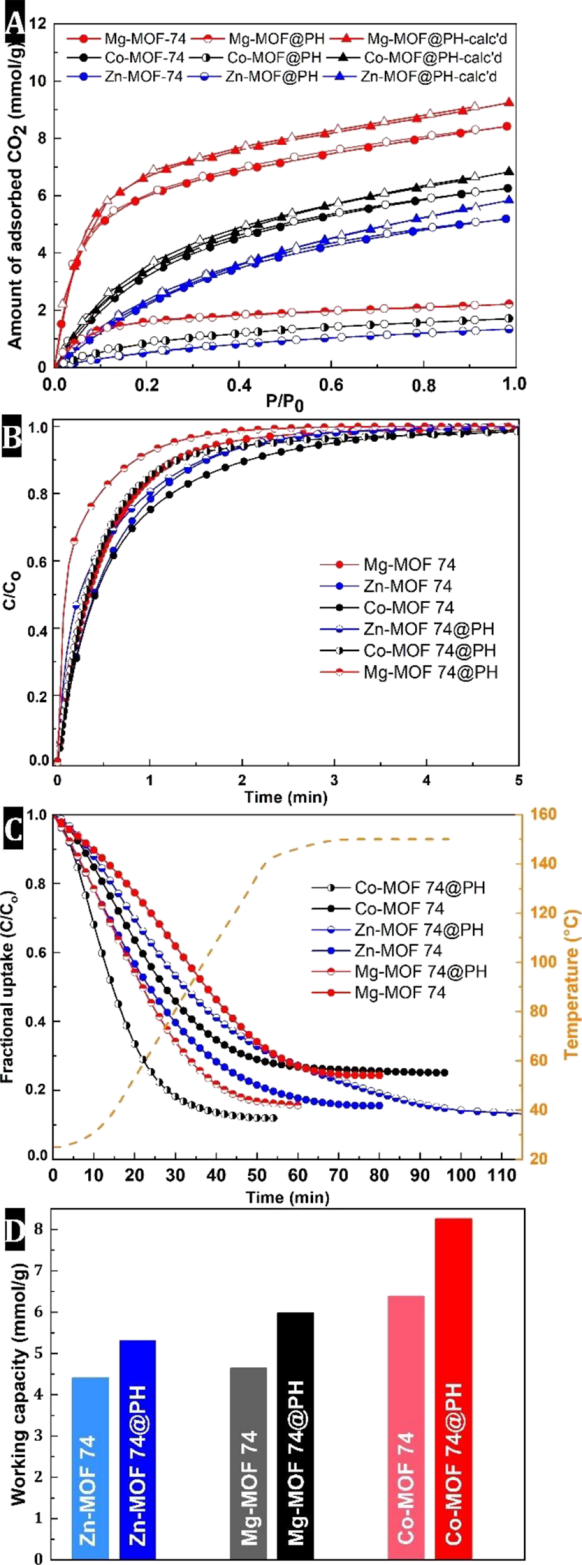
(A) CO_2_ isotherms of MOF-74 powders and MOF-74 PH composites (triangles represent calculated values of PH composites based on MOF content, full and half-full symbols: adsorption points and empty symbols: desorption points); (B) adsorption kinetics; (C) desorption kinetics during heating (dashed line indicates heating ramp); and (D) working capacities.

PH composites were tested for their potential in CO_2_ uptake at 25 °C and up to a pressure of 1 bar. CO_2_ adsorption, sorption kinetics, regeneration capabilities, and related working capacities of composites were investigated and compared with the parent MOF-74 powders. In all cases, CO_2_ adsorption capacity increases continuously with feed pressure without saturation up to 1.0 bar, clearly indicating that even larger CO_2_ uptake can be expected with further increases in pressure (Figure 3A). However, different reference materials were first investigated for their CO_2_ uptake capacities: (i) parent MOF-74 powders, (ii) PDCPD PH matrix, and (iii) MO–PH composite materials. The adsorption capacities of parent MOF-74 powders were 5.2, 8.4, and 6.3 mmol g^–1^ for Zn-, Mg-, and Co-based MOF-74, respectively, and agree well with the literature data.^42^ On the other hand, the PDCPD PH material, deprived of MOF-74 phase in the structure, and the PH composites containing MO show very low CO_2_ uptake, only about 0.05 and 0.25 mmol g^–1^, respectively (Figure S11). Finally, CO_2_ uptake of MOF-based PH composites containing Zn-, Mg-, and Co-based MOF-74 were 1.3, 2.2, and 1.7 mmol g^–1^, respectively. Since the PDCPD PH matrix and the PH composites containing only MO have very low CO_2_ uptake, it is clear that the amounts of CO_2_ adsorbed in the MOF-based PH composites are mainly due to the immobilized MOF phase. When the CO_2_ uptake values of the MOF–PH composites were calculated to the amount of MOF phase, the actual uptake was 5.8, 9.3, and 6.8 mmol g^–1^, for Zn-, Mg-, and Co-based MOF–PH composites, respectively. Adsorption kinetics is often considered an even more important parameter than working capacity in CCSU applications, as it contributes decisively to the overall performance of the adsorbent in continuous processes. Looking at the kinetic curves (Figure 4B), we can observe that the initial CO_2_ adsorption in the MOF–PH composites is much faster compared to the net MOF-74 powders, while saturation is then reached at about the same times in both systems. The reason for the better CO_2_ adsorption performance, i.e., uptake and kinetics, of the PH composite system probably lies in its easily accessible hierarchical architecture (macro-microporous). In particular, the in situ MO-to-MOF secondary recrystallization at the MO–polymer interface (so-called heterogeneous nucleation) resulted in a much smaller MOF size (about 5 μm) homogeneously distributed over the entire macropore structure within the PH that is not agglomerated and thus blocked (see SEM Figure 2D–F). Conversely, the net MOF powders are much larger and form agglomerates with a size of 100 μm, which, to some extent, closes the access to the microporous MOF channels within the agglomerates (Figure S12). This then poses a problem for gas diffusion, reducing CO_2_ uptake and impairing adsorption kinetics.

Next, temperature swing adsorption (TSA) was used to regenerate the adsorbents (Figure 4C). Regeneration by heating to 150 °C with a ramp of 5 °C/min recovered between 75 and 84% of the total adsorption capacity for Co-, Mg-, and Zn-based MOF-74 powders and between 85 and 88% for MOF–PH composites. The Zn-based MOF–PH composite material shows only a slight improvement in regeneration (86.5%) with slower desorption kinetics compared to the powdered MOF analog. On the other hand, the regeneration processes are significantly improved for the remaining two composites, with 84.2 and 88.0% of captured CO_2_ released in the case of the Mg- and Co-based MOF–PH composites, respectively. In these cases, desorption is also faster compared to the corresponding pure MOF powders. The sorption kinetics appear to be strongly dependent on the size of the MOF crystallites formed in the polymer matrix. As can be seen in Figure S12, the embedded Co- and Mg-MOF-74 have significantly smaller crystallites than those in the Zn-based MOF–PH composite, where the MOF crystals fill most of the polyHIE’s void space. However, both regeneration capacity and desorption kinetics were better in PH composites, due to the structural reasons described above. Based on the regeneration efficiencies, working capacities were estimated. The latter is defined as the difference between the total adsorption capacity and the amount of CO_2_ that remains adsorbed after the regeneration process. The working capacity represents the actual amount of CO_2_ adsorbed during the adsorption–desorption cycle and thus provides a much more representative assessment of the adsorbent’s removal capabilities than the absolute CO_2_ uptake. The PH composites showed an improvement in CO_2_ uptake under working conditions between 20 and 30% compared to the parent MOF-74 powders (Figure 4D). All PH composites also promise high durability under working conditions, as the adsorption/regeneration process in the case of the Mg-MOF-74–PH composite shows a negligible loss (0.5%) of adsorption uptake capacities after five adsorption/desorption cycles (Figure S13), demonstrating high structural stability and constant capture performance during the regeneration process.

## 4 Conclusions

Our study reports the synthesis and CO_2_ capture behavior of highly porous and co-continuous MOF-74–PH composites. The synthesis strategy combines Pickering HIPE polymerization with in situ MO-to-MOF secondary recrystallization. Pickering PHs were based on PDCPD and ZnO, Co_3_O_4_, or MgO combinations that served as substrates and precursors for composite formation. The MO-to-MOF secondary recrystallization occurred at the MO–polymer interface, where the DHBDC ligand reacted hydrothermally with the immobilized MO precursor, resulting in MOF growth (recrystallization yield of about 77%) that homogeneously filled or coated the macropores through the PH framework. The primary macropore morphology of the PHs was not affected by the integration of the MOF phase, and the resulting hierarchically porous system exhibited pronounced macro-microporosity, which is advantageous for high MOF accessibility, since about 87% of the total micropore volume is available for gases.

These highly porous MOF-74–PH composites have demonstrated their potential for applications such as CO_2_ capture: (i) higher CO_2_ adsorption capacities (about 10%) than the powdered MOF-74 analogues considering the weight fraction of net MOF-74 phase in the composites; (ii) faster adsorption kinetics and regeneration efficiency, resulting in between 20 and 30% better CO_2_ uptake under working conditions; (iii) the durability of the adsorption/regeneration process in the case of the Mg-MOF-74–PH composite shows a negligible loss (0.5%) of adsorption uptake capacities after five adsorption/desorption cycles using the temperature swing adsorption process. The enhanced CO_2_ uptake performance of MOF-74–PH composites is the result of a well-defined macrostructure created by the synthesis strategy described here. We believe that this in situ MO-to-MOF secondary recrystallization within the Pickering polyHIPEs demonstrates the ease of design and development of innovative highly porous MOF–polymer composites that will likely initiate further MOF/polymer combinations and applications in sustainable processes, such as thermal energy storage, water remediation, or catalysis to convert CO_2_ into valuable chemicals.

## Supporting Information Available

Details of material synthesis, TGA, XRD, SEM images, recrystallization efficiency calculations, and CO_2_ isotherms (PDF)
