# Supplementary material for: Metal Oxide-Derived MOF-74 Polymer Composites through Pickering Emulsion-Templating: Interfacial Recrystallization, Hierarchical Architectures, and CO2 Capture Performances
Source: ACS Appl Mater Interfaces. 2023 Mar 30;15(14):18354–61. doi: 10.1021/acsami.3c01796 (PMC10103051; doi:10.1021/acsami.3c01796)
Supplement: Supplementary file 1 — am3c01796_si_001.pdf [file am3c01796_si_001.pdf]

Supporting Information

Metal Oxide-derived MOF-74 Polymer Composites through Pickering Emulsion-Templating: Interfacial recrystallization, Hierarchical architectures and CO_2 capture performances

Nika Vrtovec,[1] Sarah Jurjevec,[1] Nataša Zabukovec Logar,[1,2] Matjaž Mazaj,[1*] Sebastijan Kovačič,[1*]

[1]National Institute of Chemistry, Hajdrihova 19, 1000 Ljubljana, Slovenia; email: sebastijan.kovacic@ki.si

[2]University of Nova Gorica, Vipavska 13, 5000 Noca Gorica, Slovenia

1. Syntheses

Materials. Zinc(II) nitrate hexahydrate ($Zn(NO_3)_2.6H_2O$, Sigma-Aldrich, 99 %), zinc oxide (ZnO, Sigma-Aldrich, 97 %), cobalt(II) acetate tetrahydrate ($Co(ac)_2.4H_2O$, Honeywell Fluka, 99 %), cobalt(II,III) oxide (Co_3O_4, Aldrich, 99.5 %), magnesium nitrate hexahydrate ($Mg(NO_3)_2.6H_2O$, Sigma-Aldrich, 99 %), magnesium oxide (MgO, Sigma-Aldrich, 95 %), 2,5-dihydroxyterephthalic acid (DHBDC, Sigma-Aldrich, 98 %), methanol (MeOH, Honeywell, 99.9 %), 2-prophanol (iPrOH, Merck, 99.9 %), ethanol (EtOH, Merck, 99.9 %), tetrahidrofuran (THF, Sigma-Aldrich, 99.9 %), *N,N'*-dimethylformamide (DMF, Sigma-Aldrich, 99.9 %), acetone (AcO, Gram-Mol, 99.5 %), oleic acid (OA, Sigma Aldrich, ≥99%), dicyclopentadiene (DCPD, Aldrich, > 96 %), Pluronic® L121 (Aldrich), toluene (Honeywell, ≥ 99.9 %), initiator M2 (Umicore) and carbon dioxide (CO_2, Messer, 99.999 %) were used as purchased without further purification.

M^{2+}-derived MOF-74 powders

Zn-MOF-74[1]: 0.5 g of $Zn(NO_3)_2.6H_2O$ (1.6 mmol) was dissolved in 1 ml of deionized water. After 15 minutes of continuous stirring, a solution containing 0.1 g of DHBDC (0.5 mmol) dissolved in 20 ml of DMF was added. The reaction mixture was then transferred to the glass vial and heated at 100 °C for 20 h. After decanting the hot mother liquor and rinsing with DMF, the product was immersed in 30 ml of MeOH, which was replaced with fresh solvent every day, for three days. The final product was then immersed in MeOH until further research was performed.

Co-MOF-74[2]: 0.3 g of $Co(ac)_2.4H_2O$ (1.5 mmol) was dissolved in 3.4 ml of deionized water. After 15 minutes of continuous stirring, 0.1 g of DHBDC (0.5 mmol) dissolved in 6.7 ml of THF was added. The reaction mixture was then transferred into a Teflon-lined autoclave and heated at 110 °C for 72 h. After cooling down to room temperature, the Co-MOF-74 was obtained by filtration. To extract solvent molecules from the pores, Co-MOF-74 was immersed in 30 mL of methanol, which was replaced daily with fresh solvent, for three days. The final product was then filtrated and dried at ambient conditions.

Mg-MOF-74[3]: 0.03 g of DHBDC (0.2 mmol) was dissolved in a mixture of 5.5 ml of MeOH and 4.5 ml of DMF. After 10 minutes of continuous stirring, 0.1 g of $Mg(NO_3)_2.6H_2O$ (0.4 mmol) was added to the solution. The reaction mixture was then transferred into a Teflon-lined autoclave and heated at 120 °C for 22 h. The reaction mixture was decanted and the final product was soaked three times with DMF and MeOH (1:1). The final product was stored in MeOH prior the further studies.

Metal oxide (MO)-derived MOF-74 powders

ZnO-MOF-74: 0.1 g of DHBDC (0.5 mmol) was dissolved in a mixture of 10 ml DMF, 0.5 ml iPrOH and 0.5 ml of deionized water. After 10 minutes of continuous stirring, 0.1 g of ZnO (1.0 mmol) was added to the solution. The reaction mixture was then transferred to the glass vial and heated at 100 °C for 20 h. The final product was then filtrated, washed with DMF and dried at ambient conditions.

Co_3O_4-MOF-74: 0.1 g of DHBDC (0.5 mmol) was dissolved in a mixture of 6.7 ml of THF and 3.3 ml of deionized water. After 10 minutes of continuous stirring, 0.1 g of Co_3O_4 (0.3 mmol) was added to

the solution. The reaction mixture was then transferred to the glass vial and heated at 110 °C for 72 h. The final product was then filtrated, washed with THF and dried at ambient conditions.

MgO-MOF-74: 0.03 g of DHBDC (0.2 mmol) was dissolved in a mixture of 9.0 ml of DMF, 0.6 ml of EtOH and 0.6 ml of deionized water. After 10 minutes of continuous stirring, 0.01 g of MgO (0.2 mmol) was added to the solution. The reaction mixture was then transferred into a Teflon-lined autoclave and heated at 120 °C for 26 h. The final product was then filtrated, washed with mixture of EtOH and DMF (1:1) and dried at ambient conditions.

Oleic acid (OA) coated nanoparticles. The surface of ZnO, Co_3O_4 and MgO nanoparticles (NPs) was modified with oleic acid (OA). Nanoparticles were suspended in the solution of OA and ethanol (the mass fraction of OA was 15 wt% to NP) by sonication. Subsequently, the suspension was mixed with magnetic stirrer for 48 h at room temperature and dried in the oven at 50 °C overnight. The amount of OA attached onto the NPs' surface was determined by TGA.

Metal oxide-based PH composite monoliths:

Water-in-oil (W/O) HIPEs were used to obtain PDCPD-based PH composites. The DCPD monomer (1.30 g), Pluronic® L121 (0.065 g), toluene (50µL) and OA-coated NPs (between 10 and 30 wt% according to the DCPD) were placed in a 3-neck round-bottomed flask equipped with a mechanical stirrer and a dropping funnel. The mixture was stirred at 400 rpm for 5 min and upon continuous stirring at 25°C deionized water (5.5 mL) was added drop-wise over about 1 h. Afterwards, the initiator M2 (1.3 mg, 0.0007 mmol in respect to DCPD**)** dissolved in toluene (0.25 mL) was added and the emulsion was stirred for further 5 min. Subsequently, the emulsion was transferred to an appropriate mould (i.e. glass vials). The filled moulds were transferred into a preheated oven operating under air. Curing of the emulsions at 80 °C for 4 h resulted in the formation of white rigid monoliths in all cases. The specimens were purified by Soxhlet extraction with acetone for 24 h and subsequently dried in a desiccator under vacuum (10 mbar) until the weight was constant.

MOF-based PH composite monoliths:

MOF-based PH composite materials were then solvothermally crystallized from metal oxide-based PH composite precursors.

Zn-MOF 74-based PH composites: For the recrystallization of ZnO-based PHs, 0.05 g DHBDC (0.3 mmol) was dissolved in the mixture of three solvents – 5.0 ml of DMF, 0.3 ml of iPrOH and 0.3 ml of deionized water. After 15 minutes of continuous stirring, a piece of ZnO-based PH monolith (0.3 g) was added to the mixture. The reaction mixture was then transferred into a Teflon-lined stainless-steel autoclave and heated at 110 °C for 48 h. After the solvothermal treatment, the recrystallized composite was rinsed with acetone and dried at ambient conditions.

Co-MOF 74-based PH composites: For the recrystallization of Co_3O_4-based PH, 0.06 g of DHBDC (0.4 mmol) was dissolved in mixture of 10 ml of DMF and 10 ml of EtOH. After 15 minutes of continuous stirring, a piece of Co_3O_4-based PH monolith (0.3 g) was added to the mixture, which was transferred into a glass vial and heated at 150 °C for 120 h. After the

solvothermal treatment, the recrystallized composite was rinsed with acetone and dried at ambient conditions.

Mg-MOF 74-based PH composites: For the recrystallization of MgO-based PH, 0.03 g of DHBDC (0.2 mmol) was dissolved in a mixture of 4.5 ml of DMF and 5.5 ml of MeOH. After 15 minutes of continuous stirring, a piece of MgO-based PH monolith (0.3 g) was added to the mixture. The reaction mixture was then transferred into a Teflon-lined stainless-steel autoclave and heated at 150 °C for 48 h. After the solvothermal treatment, the recrystallized composite was rinsed with acetone and dried at ambient conditions.

2. Thermogravimetric analysis (TGA) of MO

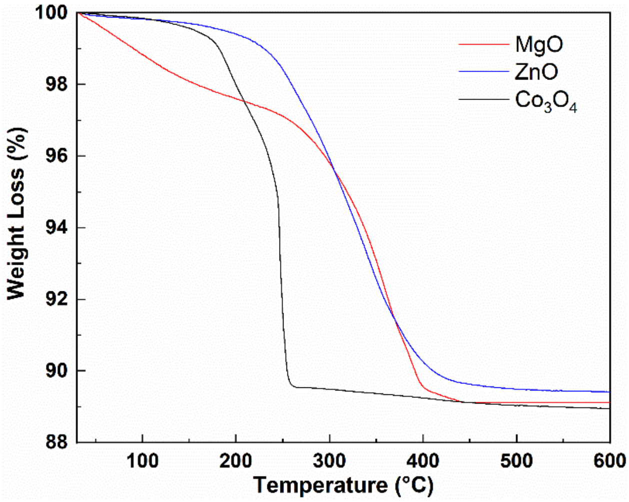

Figure S1. TGA of the MO after surface modification by OA

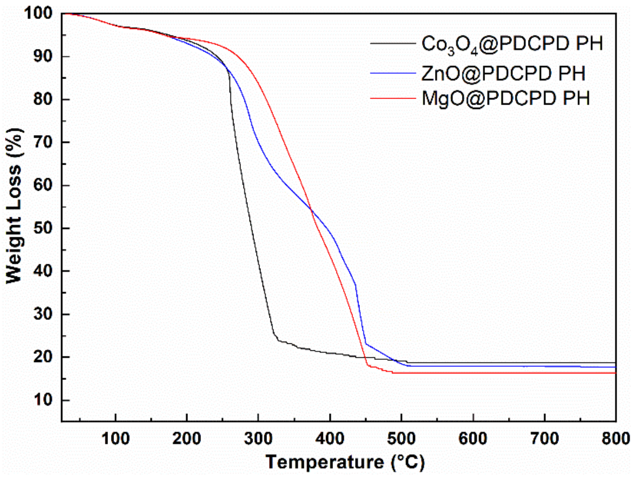

Figure S2. TGA of the MO@PDCPD PHs

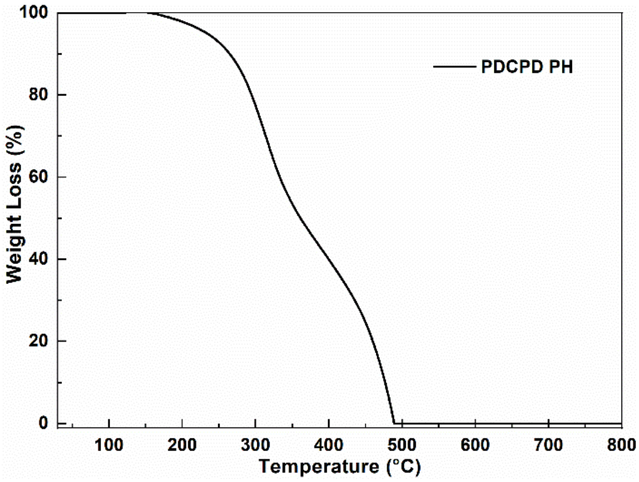

Figure S3. TGA of the PDCPD PHs

3. XRD analysis of MOF 74@ PH composites

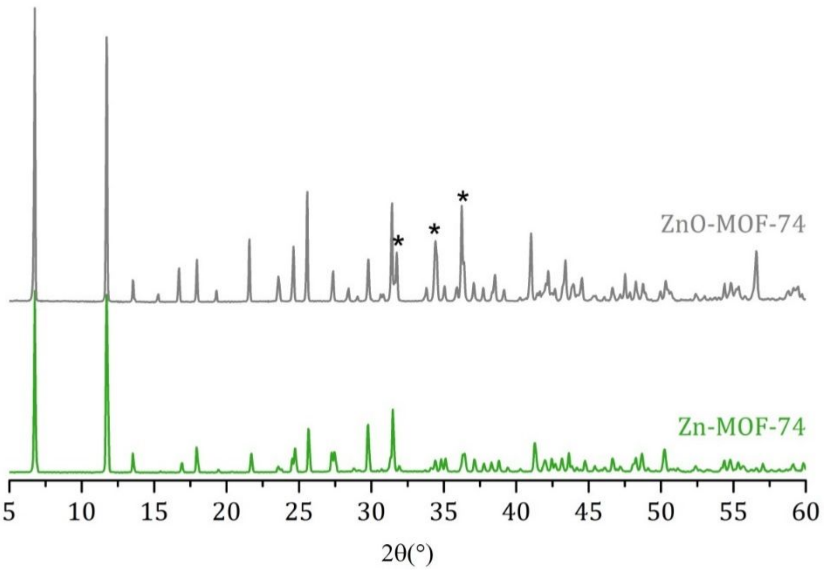

Figure S4. XRD patterns of the referenced powdered Zn-MOF-74 prepared according to N.L. Rosi et al.[1] and the product synthesized from ZnO precursor (ZnO-MOF-74). The latter product contains 25 wt.% of ZnO according to Rietveld quantitative phase analysis. Reflections corresponding to unreacted ZnO phase are indicated by asterisks.

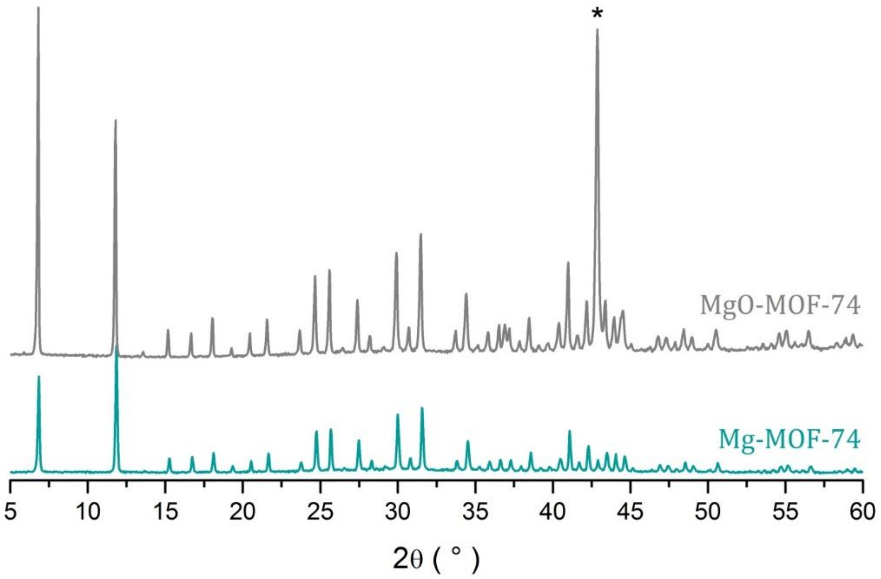

Figure S5. XRD patterns of the referenced powdered Mg-MOF-74 prepared according to T.M. McDonald et al.[3] and the product synthesized from MgO precursor (MgO-MOF-74). The latter product contains 49 wt.% of MgO according to Rietveld quantitative phase analysis. Reflection corresponding to unreacted MgO phase is indicated by asterisk.

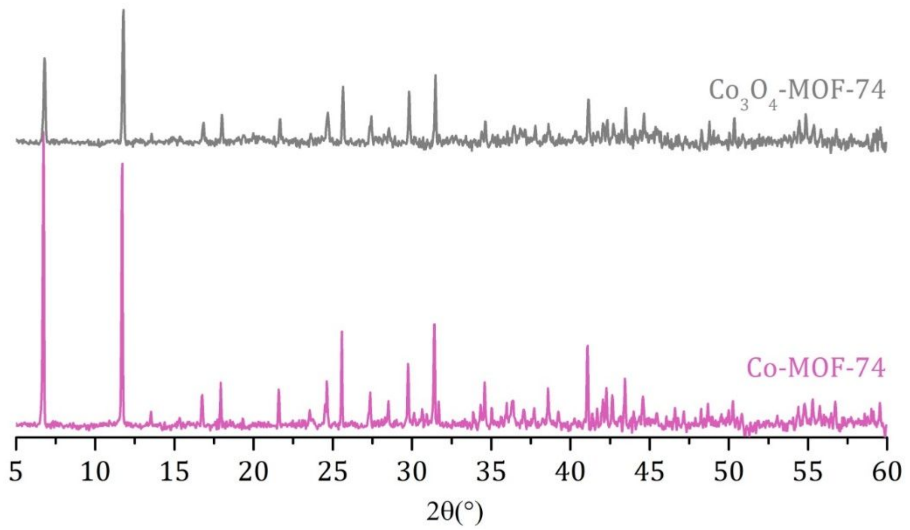

Figure S6. XRD patterns of the referenced powdered Co-MOF-74 prepared according to P. Dietzel et al.[2] and the product synthesized from Co_3O_4 precursor (Co-MOF-74). There are no visible reflections of Co_3O_4 in the pattern of Co-MOF-74 sample indicating that the recrystallization from metal-oxide to MOF-74 is complete.

4. SEM characterization of the MOF@PH composites

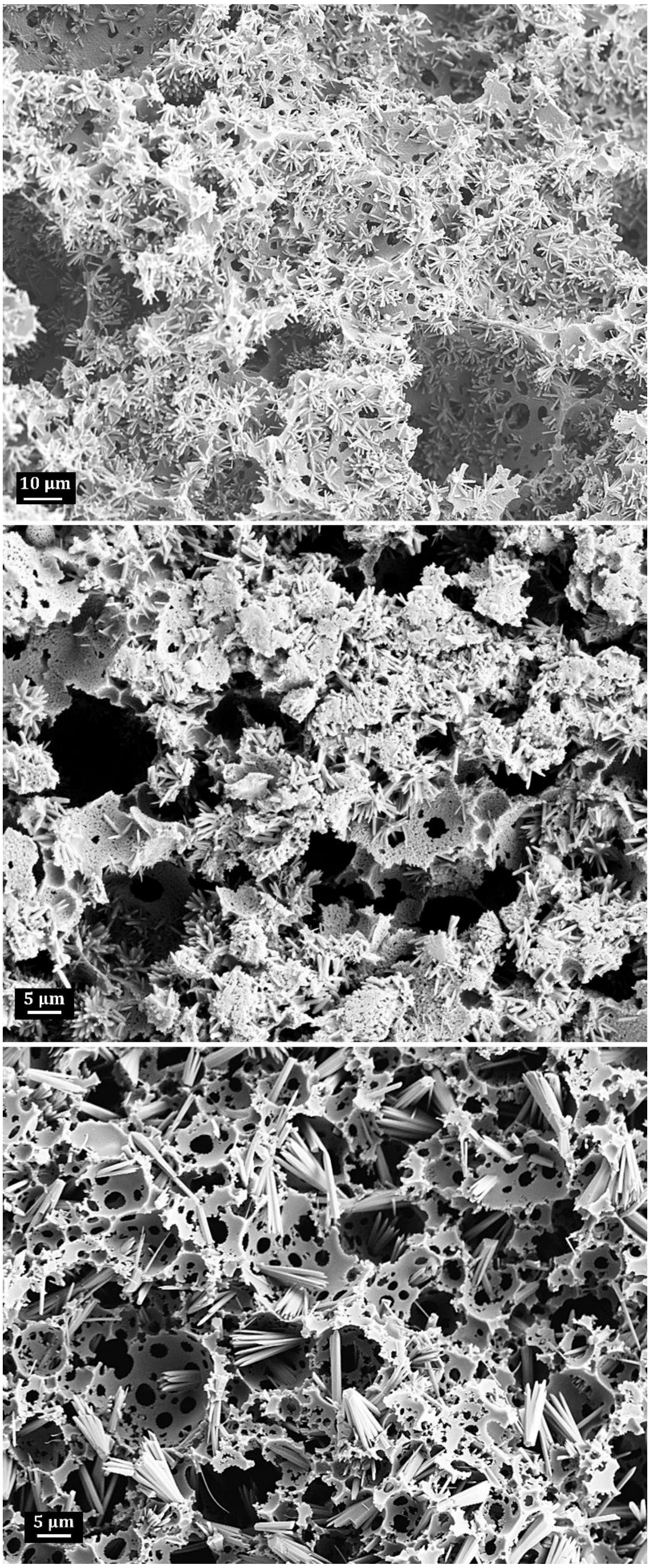

Figure S7. SEM micrographs at lower magnification of Zn-MOF@PH composite (upper); Mg-MOF@PH composite (middle) and Co-MOF@PH composite (bottom)

5. Calculations of MOF contribution within the composites based on the TG analysis

The calculations are dissected to several steps for clearance purposes.

Mass fraction of metal oxide within MOF-74 with formula $M_2(DOBDC)$:

$$w(MO) = \frac{2.Mr(MO)}{Mr(M_2(DOBDC))};$$

Weight contribution of metal oxide within MO@polyHIPE composites according to TGs excluding solvent content:

$$w(MO - com1) = \frac{wt\,\%\, residue\ from\ MO@polyHIPE\ TG}{1 - 100/wt\%\ of\ solvent\ from\ MO@polyHIPE\ TG};$$

Weight contribution of metal oxide within MOF74@polyHIPE composites according to TGs excluding solvent content:

$$w(MO - com2) = \frac{wt\,\%\, residue\ from\ MOF74@polyHIPE\ TG}{1 - 100/wt\%\ of\ solvent\ from\ MOF74@polyHIPE\ TG};$$

Weight contribution of inert polyHIPE matrix within MO@polyHIPE composite:

$$W_{poly} = 100 - w(MO - com(1{,}2);$$

Theoretical weight contribution of MOF-74 within MOF-74@polyHIPE composite if all oxide is recrystallized into MOF:

$$w1(MOF74) = \frac{w_{MO-com1}/w_{MO}}{w_{MO-com1}/w_{MO} + W_{poly}};$$

Calculated weight contribution of MOF-74 within MOF-74@polyHIPE composite:

$$w2(MOF74) = \frac{w_{MO-com2}/w_{MO}}{w_{MO-com2}/w_{MO} + W_{poly}};$$

Recrystallization yield:

$$\frac{w_2(MOF74)}{w_1(MOF74)}$$

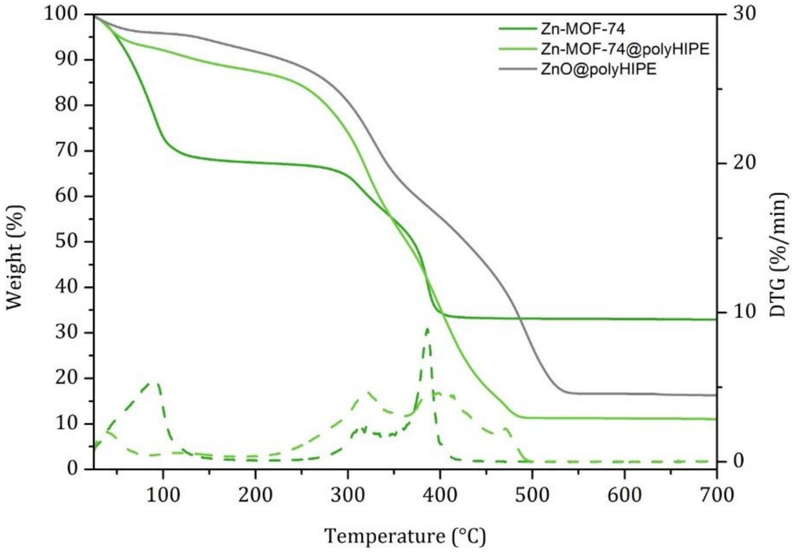

Figure S8. TG (full lines) and DTG (dashed lines) curves of ZnMOF-74@polyHIPE, ZnO@polyHIPE composites and referenced ZnMOF-74 powder.

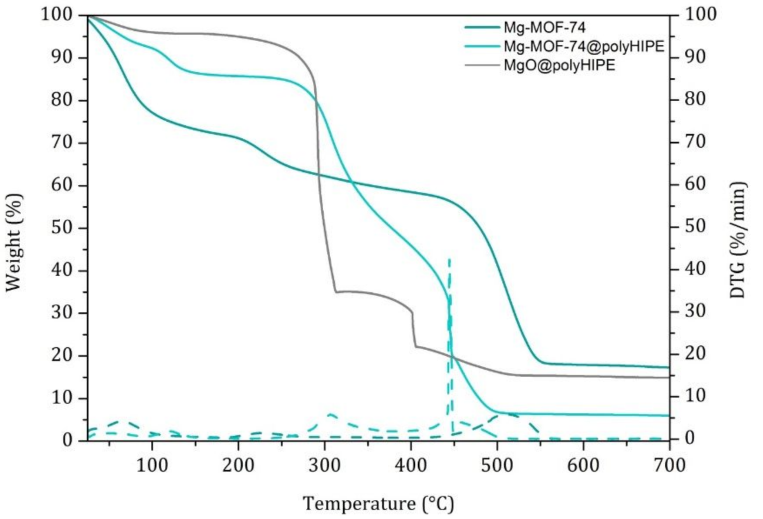

Figure S9. TG (full lines) and DTG (dashed lines) curves of MgMOF-74@polyHIPE, MgO@polyHIPE composites and referenced MgMOF-74 powder.

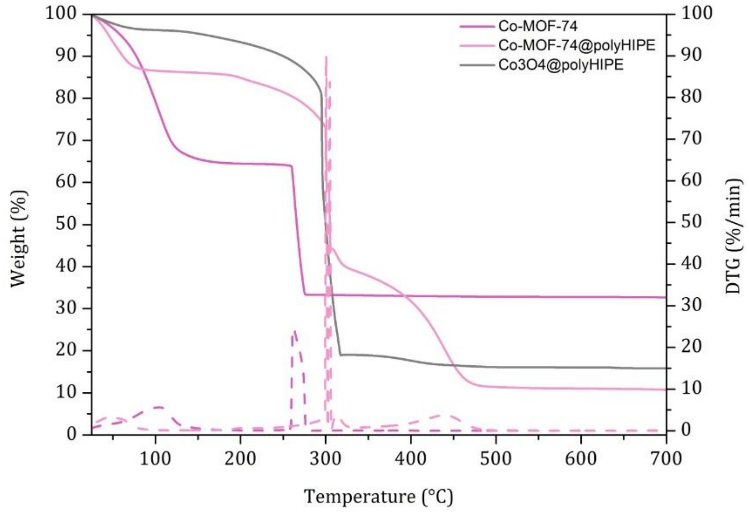

Figure S10. TG (full lines) and DTG (dashed lines) curves of CoMOF-74@polyHIPE, CoO@polyHIPE composites and referenced CoMOF-74 powder.

6. CO_2 adsorption tests

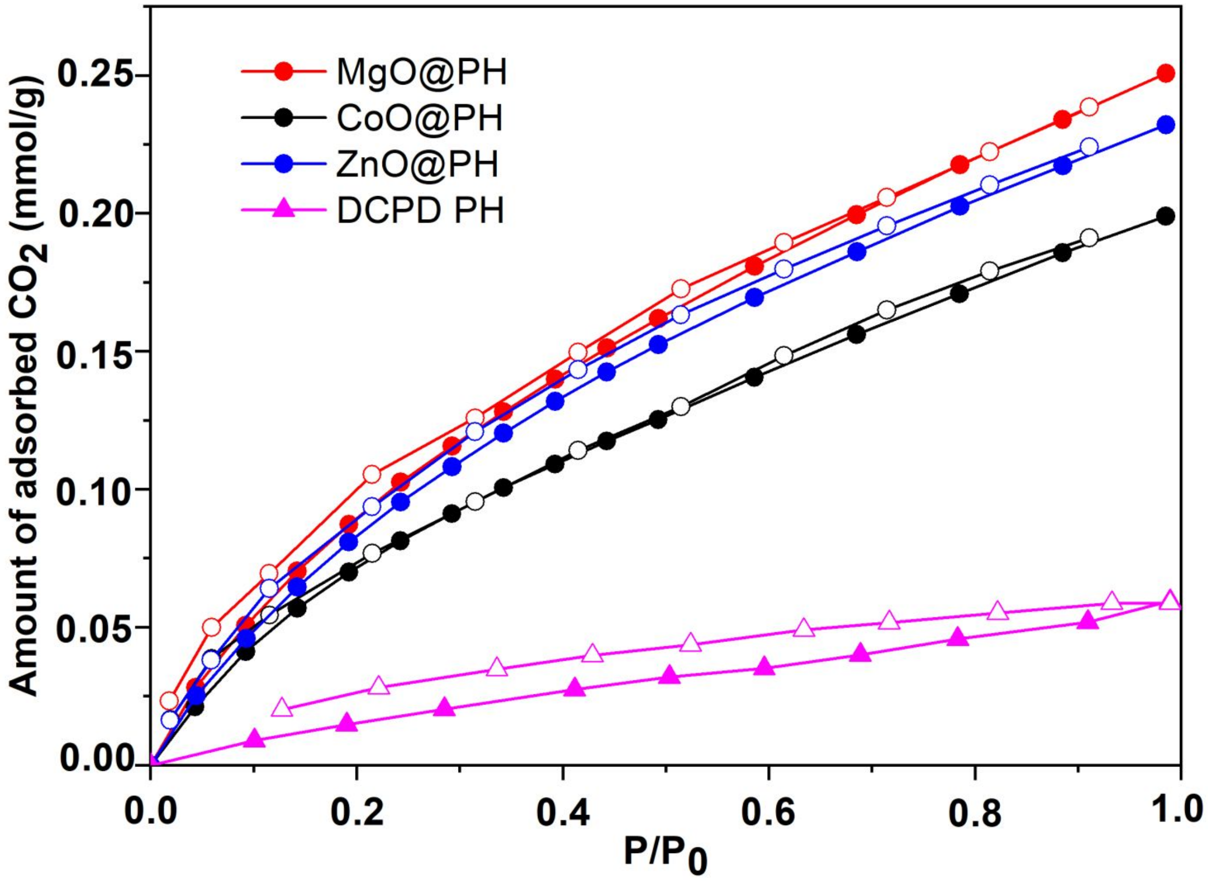

Figure S11: CO_2 isotherms of MO@PH composites and PDCPD PH material refernced materials measured at 25 °C.

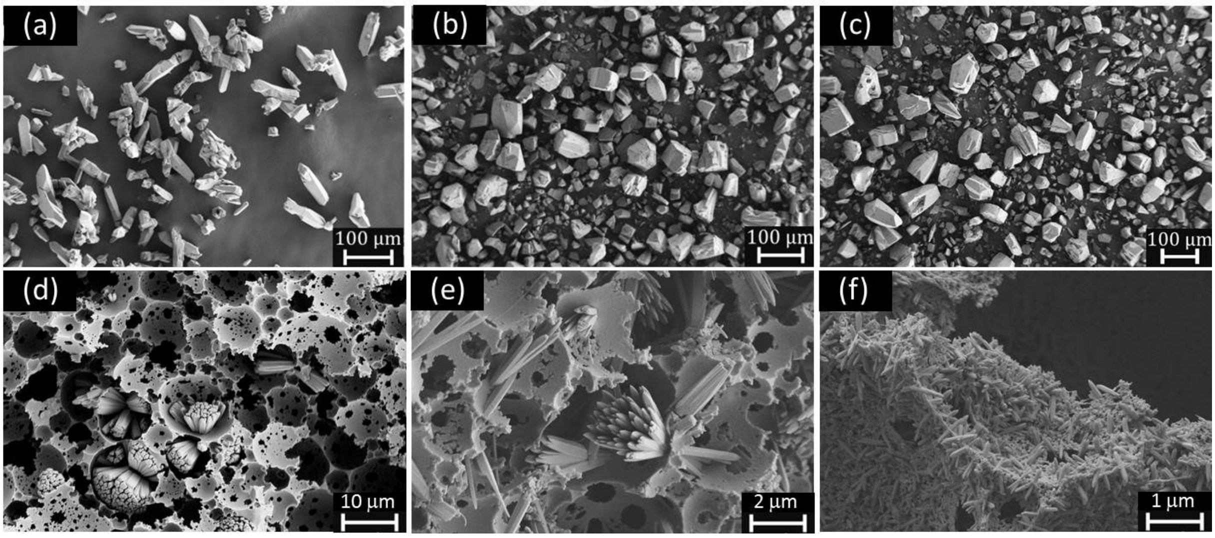

Figure S12. SEM micrographs of (a) Zn-MOF-74, (b) Co-MOF-74, (c) Mg-MOF-74, (d) Zn-MOF-74@polyHIPE, (e) Co-MOF-74@polyHIPE and (f) Mg-MOF-74@polyHIPE products. Pristine MOF-74 powders exhibit crystals with the estimated size up to 100 µm. Corresponding MOF-74 crystallites imbedded within the polyHIPE matrices have dimensions from submicron range to 5 µm.

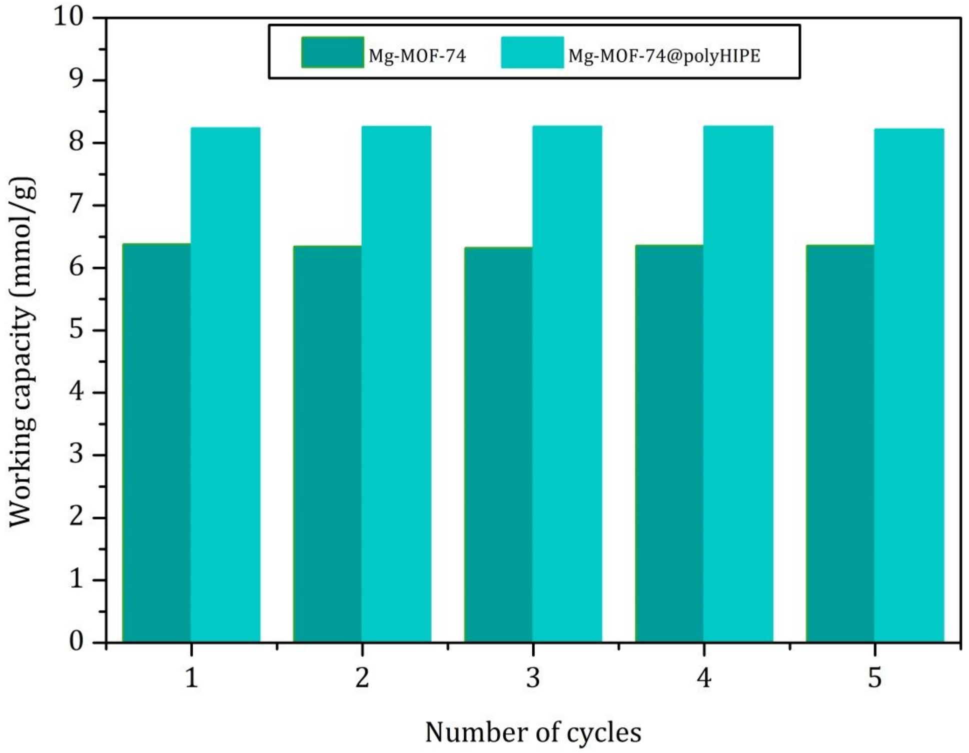

Figure S13. Working capacities for powder Mg-MOF-74 and Mg-MOF-74@polyHIPE composite during 5 adsorption/desorption cycles.

References:

1) Rosi, N. L.; Kim, J.; Eddaoudi, M.; Chen, B.; O'Keeffe, M.; Yaghi, O. M. Rod Packings and Metal−Organic Frameworks Constructed from Rod-Shaped Secondary Building Units. *J. Am. Chem. Soc.* **2005**, *127* (5), 1504–1518. https://doi.org/10.1021/ja045123o.

(2) Dietzel, P. D. C.; Morita, Y.; Blom, R.; Fjellvåg, H. An In Situ High-Temperature Single Crystal Investigation of a Dehydrated Metal-Organic Framework Compound and Field-Induced Magnetization of One-Dimensional Metal-Oxygen Chains. *Angew. Chemie Int. Ed.* **2005**, *44* (39), 6354–6358. https://doi.org/10.1002/anie.200501508.

(3) McDonald, T. M.; Mason, J. A.; Kong, X.; Bloch, E. D.; Gygi, D.; Dani, A.; Crocellà, V.; Giordanino, F.; Odoh, S. O.; Drisdell, W. S.; Vlaisavljevich, B.; Dzubak, A. L.; Poloni, R.; Schnell, S. K.; Planas, N.; Lee, K.; Pascal, T.; Wan, L. F.; Prendergast, D.; Neaton, J. B.; Smit, B.; Kortright, J. B.; Gagliardi, L.; Bordiga, S.; Reimer, J. A.; Long, J. R. Cooperative Insertion of CO2 in Diamine-Appended Metal-Organic Frameworks. *Nature* **2015**, *519* (7543), 303–308. https://doi.org/10.1038/nature14327.
